# Case report: p.Glu134del *SOD1* mutation in two apparently unrelated ALS patients with mirrored phenotype

**DOI:** 10.3389/fneur.2022.1052341

**Published:** 2023-01-04

**Authors:** Giulia Gianferrari, Ilaria Martinelli, Cecilia Simonini, Elisabetta Zucchi, Nicola Fini, Serena Carra, Cristina Moglia, Jessica Mandrioli

**Affiliations:** ^1^Department of Biomedical, Metabolic and Neural Sciences, University of Modena and Reggio Emilia, Modena, Italy; ^2^Department of Neurosciences, Azienda Ospedaliero-Universitaria di Modena, Modena, Italy; ^3^Clinical and Experimental PhD Program, University of Modena and Reggio Emilia, Modena, Italy; ^4^Neurosciences PhD Program, University of Modena and Reggio Emilia, Modena, Italy; ^5^S.C Neurology 1U, Azienda Ospedaliero-Universitaria Città della Salute e della Scienza Torino, Torino, Italy; ^6^“Rita Levi Montalcini” Department of Neuroscience, University of Turin, Torino, Italy

**Keywords:** ALS, genotype-phenotype correlation, p.Glu134del *SOD1*, case report, motor neuron diseases

## Abstract

With upcoming personalized approaches based on genetics, it is important to report new mutations in amyotrophic lateral sclerosis (ALS) genes in order to understand their pathogenicity and possible patient responses to specific therapies. *SOD1* mutations are the second most frequent genetic cause of ALS in European populations. Here, we describe two seemingly unrelated Italian patients with ALS carrying the same *SOD1* heterozygous c.400_402 deletion (p.Glu134del). Both patients had spinal onset in their lower limbs, progressive muscular weakness with respiratory involvement, and sparing bulbar function. In addition to the clinical picture, we discuss the possible pathogenic role of this unfamiliar *SOD1* mutation.

## Introduction

First identified by Rosen et al. (1), the *SOD1* gene has become significant in amyotrophic lateral sclerosis (ALS) genetics, representing the second most frequent gene involved in ALS European cohorts, after *C9ORF72* (2), and accounting for ~18–20% of familial (fALS) and 1–2% of sporadic (sALS) cases (3). To date, more than 180 *SOD1* mutations have been associated with ALS (4).

Various mutations in *SOD1* differentially influence disease phenotypes across a broad spectrum of manifestations (4). In particular, data from the ALSoD database^1^ show that ~95% of *SOD1*-ALS patients have spinal onset in the lower limbs (5) with predominant lower motor neuron (LMN) involvement (6), a mean age of 48 years at onset, and no significant gender predominance. Increasing evidence from clinical cases and population studies has revealed that *SOD1*-ALS phenotypes can vary depending on the mutations (2, 7). Some of these, such as the A4V, H43R, L84V, G85R, N86S, and G93A mutations, are associated with an aggressive form of ALS with short survival rates; others, including G93C, D90A, and H46R, are associated with longer survival rates (7).

Although the predominant symptoms depend on LMN involvement, manifestations of upper motor neurons (UMN) may also be present, as reported in a systematic review by Connolly et al. (2). Additionally, the presence of pathological motor-evoked potentials (MEPs) with prolonged central conduction latencies has been described in patients with different *SOD1* mutations (8).

In recent decades, increasing literature data have revealed a great clinical variation in *SOD1*-ALS patients, encompassing extra-motor symptoms (4), and widening the one-to-one correspondence (2). Moreover, the demonstration of the pathogenic role of single variants is increasingly crucial because of the therapeutic intervention to prevent *SOD1* synthesis and accumulation (9, 10) by the intrathecally administered antisense oligonucleotide, Tofersen (10).

In this regard, the dysfunctional effect or evidence of familial segregation of a single *SOD1* variant could play a key role in pathogenicity validation and counseling in clinical practice (11).

Here, we describe the phenotypic and genotypic features of two seemingly unrelated Italian patients with ALS carrying a rare mutation in *SOD1*.

## Case report

### Case 1

**Figure 1:**
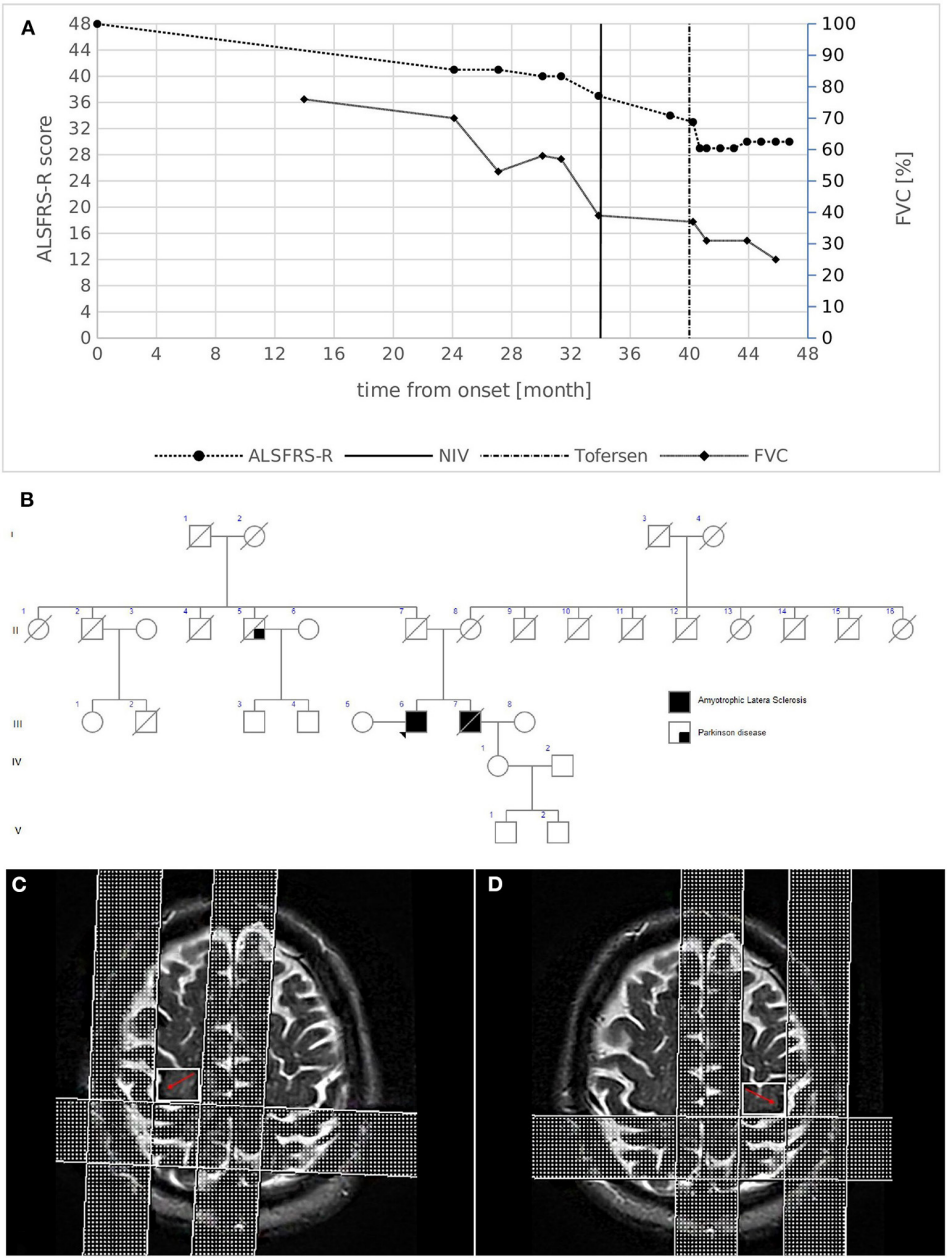
**(A)** Disease course of case 1. The graph shows ALSFRS-R total score (left) and FVC % (right) decline from disease onset. The bold vertical line represents the time when NIV was initiated, while the dashed vertical line represents the beginning of intrathecal Tofersen administration. **(B)** Family pedigree of case 1. Filled circle/square, affected individuals; open circle/square, unaffected individuals; arrow, proband; diagonal line, deceased individuals. The proband's mother died at 79 years of age from HCV infection complications and chronic COPD; the father died at 94 years of age after a hip fracture. A paternal uncle died at an advanced age because of Parkinson's disease. **(C,D)** Brain MRI images of case 1. MRI T2-weighted images revealing the hypointensity along the bilateral motor cortex (red arrows).

A 61-year-old male presented with a 2-year history of progressive distal weakness of the lower limbs. His medical and social history were uninformative. He reported a family history of ALS (Figure 1B): his brother died at 59 years of age because of ALS, characterized by progressive weakness in the lower limbs with respiratory impairment leading to non-invasive ventilation (NIV) 5 years after onset. Neurological examination of the patient revealed moderate weakness in both lower limbs with hypotrophy, whereas the upper limb and bulbar function were initially spared. Widespread fasciculations involved all four limbs. Deep tendon reflexes were brisk in all limbs, more pronounced on the left side, and without spasticity. After an extensive diagnostic work-up, including neurophysiological studies that revealed active denervation and reinnervation potentials in three body regions, the diagnosis of ALS was established based on the revised El Escorial Criteria. Interestingly, MRI revealed UMN involvement as hypointensity along the bilateral motor cortex on T2-weighted images (Figures 1C, D), with a reduction in the fractional anisotropy (FA) values of the pyramidal bundle in tractography acquisitions.

Reduced amplitudes with normal latencies and central motor conduction times were observed in the lower limbs, as measured by transcranial MEPs.

Extensive neuropsychological testing revealed normal cognitive profiles. Respiratory function showed asymptomatic initial impairment [forced vital capacity (FVC):70%], and the revised ALS functional rating scale (ALSFRS-R) score was 41/48 (progression rate: 0.29 points/month). One year later, the weakness had spread to the upper limbs, and NIV was initiated to support respiratory function (Figure 1A). The patient remains alive 48 months after the onset of symptoms. He currently demonstrates weakness in four limbs with predominant LMN signs, respiratory impairment (FVC 25%) without bulbar involvement, and is undergoing treatment with intrathecal Tofersen (early access program).

### Case 2

**Figure 2:**
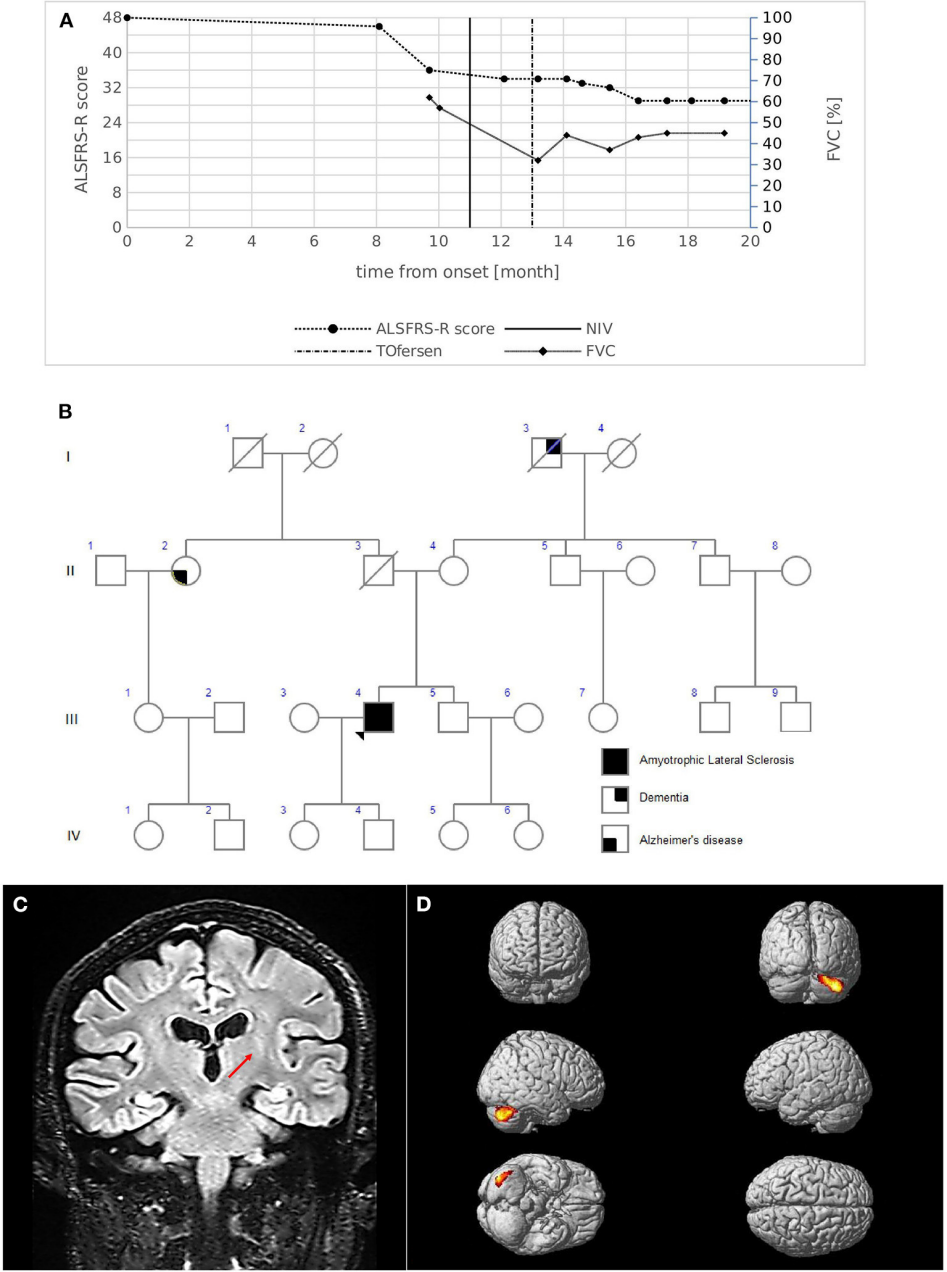
**(A)** Disease course of case 2. The graph shows ALSFRS-R total score (left) and FVC % (right) decline from the onset of symptoms. The bold vertical line represents the time when NIV was initiated, while the dashed vertical line represents the beginning of intrathecal Tofersen administration. **(B)** Family pedigree of case 2. Filled circle/square, affected individuals; open circle/square, unaffected individuals; arrow, proband; diagonal line, deceased individuals. The proband's father died at 56 years of age of colon cancer; the mother is still alive; a 51-year-old brother is alive and in good health and is currently undergoing psychological counseling to evaluate genetic testing. Maternal grandfather had dementia which started at an advanced age; a paternal aunt had Alzheimer's disease with onset at 75 years. **(C)** MRI coronal acquisition showing FLAIR hyperintensity of left cortico-spinal bundle; the red arrow indicates the peculiar involvement of the internal capsule segment. **(D)** ^18^FDG-PET images Glass brain rendering of the comparison between subject 2 and 40 healthy controls (HC). The cluster showing a statistically significant relative hypermetabolism in subject 2 compared to HC is projected on the brain surface (height threshold *p* < 0.001; *p* < 0.05 FWE-corrected at cluster level). For ^18^F-FDG-PET acquisition and elaboration details please refer to Canosa et al. (12) and Supplementary material.

A 53-year-old male presented with an 8-month history of walking difficulties and upper-limb proximal weakness. His medical history included essential tremor (ET), bipolar disorder, asthma, and diverticular disease. Neurological examination showed mild proximal weakness in the upper limbs and mild weakness in the lower limbs, mainly on the right side, with hypotrophy of the intrinsic hand muscles and the right calf. Bulbar function was completely unaffected. Deep tendon reflexes were brisk in all limbs with bilateral Hoffman and Babinski signs. Spasticity was absent. No family history of ALS was reported (Figure 2B). Diagnostic tests led to an ALS diagnosis according to the revised El Escorial Criteria. Electrophysiological studies showed active denervation and reinnervation potentials in three body regions. Increased central motor conduction time with increased latency and reduced amplitude was observed in the right upper limb as measured by transcranial MEPs. MRI T2-weighted images showed hypointensity of the bilateral motor cortex and FLAIR hyperintensity of the left cortico-spinal bundle (Figure 2C). MRI tractography revealed reduced FA values in the cortico-spinal tract acquisitions. 18FDG-PET showed areas of hypermetabolism affecting both cerebellar hemispheres, with a prevalence in the right lobe (Figure 2D).

Respiratory symptoms emerged 11 months after onset, and NIV was initiated. The progression rate measured by ALSFRS-R revealed that monthly decline at diagnosis was slow (0.25 points/month); however, a subsequent acceleration (1.27 points/month) was present due to respiratory function decline (Figure 2A). The patient remains alive 20 months after the onset of symptoms. He currently demonstrates weakness in four limbs, respiratory impairment (FVC 45%), a predominant LMN phenotype without bulbar involvement, and is being treated with intrathecal Tofersen (early access program).

## Methods

**Figure 3:**
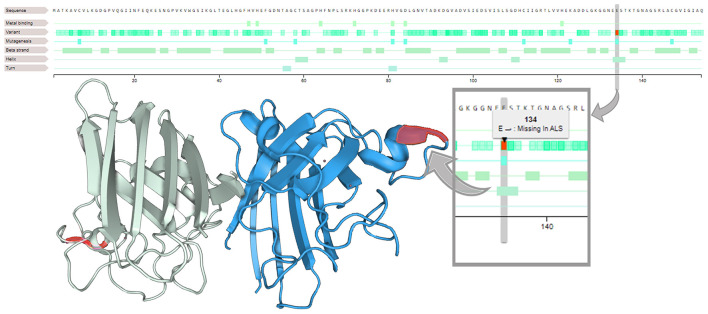
Loss of glutamic acid in position 134 (on the right). On the SOD1 3D structure (on the left), residues 133–144 form an electrostatic ring in an alpha helix segment, a loop element that is critical in the binding function of the protein.

After sample processing (see Supplementary material), an NGS probe-based customized panel was performed (13), revealing c.400_402 deletion in *SOD1* in both patients, resulting in heterozygous p.Glu134 deletion (Figure 3). The interpretation of this deletion was conducted in line with the ClinVar tools (see Supplementary material). Unfortunately, in the first case report, the proband's brother died without undergoing genetic testing, and a blood sample was not available.

## Discussion

We describe a heterozygous deletion (c.400_402) in the *SOD1* gene rarely reported previously in two unrelated patients with and without a family history of ALS, which caused an *in-frame* 3-nucleotide deletion with loss of glutamic acid at position 134 (p.Glu134del) in the *SOD1* protein. This deletion is not present in population databases (ExAC no frequency)^2^ and is classified as a Variant of Uncertain Significance (ClinVar^2^).

p.Glu134del of the *SOD1* gene translated into a polypeptide with one less amino acid than the wild-type: one glutamine instead of two would be present between positions 131 and 134, and previous experimental studies have shown that missense changes disrupt *SOD1* protein function (14, 15).

*SOD1* variants with substantial metal binding and superoxide dismutase activities, called wild-type-like *SOD1* variants (15), are one of the two classes in which the variants of *SOD1* have been classified. They can primarily cause perturbation of the *SOD1* electrostatic loop (15) involving residues 133–144 (electrostatic ring) and adjacent residues 119–132 (electrostatic circuit). Similarly, the previously described p.Glu133 deletion, which is located in an alpha-helical segment in a loop element crucial for binding, is poorly tolerated and is associated with a higher propensity to aggregate (16). This leads to increased intermolecular interactions and fibril formation, aggregation, and loss of *SOD1* stability, which represent synergistic risk factors for ALS severity (15).

Moreover, considering the new nomenclature of *SOD1* variants, p.Glu134del could be comparable with the p.Glu133del mutation described by Hosler et al. (17) and Sabatelli et al. (18), in which TDP-43 abnormalities in fibroblasts derived from the patient were found (18).

Interestingly, the pathogenic effect of this mutation could be postulated by the discovery of the same mutation in two unrelated ALS cases and by the overlapping location and function at the sequence level, since both Glu residues are encoded by the same GAA codon (18).

The clinical picture related to p.Glu133 deletion consists of a lower motor neuron predominant phenotype, with disease onset at 55 years of age and a disease duration of 82 months (18).

In our cases, morphological MRI studies and tractography data were consistent with previous literature (19, 20). The peculiar metabolic alterations in cerebellar regions found in PET images for case 2 seem to be distinct from previous reports on *SOD1*-ALS (12, 21), which, compared to healthy controls, demonstrate relative hypermetabolism in the right precentral gyrus and paracentral lobule (12), or involvement of the precentral gyrus, superior, middle, and inferior frontal gyrus, anterior cingulate, and medial part of the superior gyrus (21).

Interestingly, case 2 reported a history of ET, which could contribute to cerebellar PET alterations, since previous studies found functional and structural abnormalities in several parts of the anterior and posterior cerebellar lobules in ET (22). Additionally, in ALS literature, previous PET studies have detected hypermetabolism in various cerebellar regions (23) with different proposed biological explanations, such as microglial activation, loss of inhibition, or compensatory processes (24).

Interestingly, while both patients clinically presented with predominant LMN signs, they both had clinical signs and showed radiological and neurophysiological involvement of UMN, which is in line with recent studies suggesting widespread motor neuron involvement in *SOD1*-ALS (12).

Finally, with regard to the effect of ongoing treatment with Tofersen in the early access program^2^, both patients experienced a slowdown in disease progression after starting the treatment as measured by ALSFRS-r, and a less rapid decline or stabilization of respiratory function, as recently reported in a phase III clinical trial (VALOR and OLE) (10). The patients were treated for 8 and 7 months, respectively, and the side effects reported were those associated with lumbar puncture, such as headache and procedural pain, consistent with the literature data (10).

## Conclusions

We identified an *SOD1* p.Glu134 deletion in two seemingly unrelated Italian patients with an overlapping phenotype, characterized by a predominant LMN phenotype, with spinal onset in the lower limbs, and respiratory involvement.

The correlation between the p.Glu134del mutation and mirrored ALS phenotypes along with the molecular effects on *SOD1* stability suggest a possible pathogenic role of this mutation, becoming a possible target for *SOD1-specific* therapy. Signaling rare *SOD1* mutations and their corresponding phenotypes is gaining importance in this scientific era with the advent of personalized genetic-based approaches.

## Data availability statement

The datasets presented in this article are not readily available because of ethical and privacy restrictions. Requests to access the datasets should be directed to the corresponding author.

## Ethics statement

The studies involving human participants were reviewed and approved by Comitato Etico provinciale di Modena (number 124/08, September 2, 2008). The patients/participants provided their written informed consent to participate in this study. Written informed consent was obtained from the individual(s) for the publication of any potentially identifiable images or data included in this article.

## Author contributions

JM and IM: conceptualization. JM, IM, GG, CS, and EZ: interpreting data. GG, IM, CS, EZ, NF, and CM: recruitment. GG, IM, JM, and SC: writing—original draft preparation. JM, SC, CM, IM, and EZ: writing—review and editing. JM: funding acquisition. All authors contributed to the article and approved the submitted version.

## Footnotes

^1^ALSoD database: https://alsod.ac.uk/.

^2^ https://www.biogen.com/en_us/access-programs.html
