# Supplementary material for: Case report: p.Glu134del SOD1 mutation in two apparently unrelated ALS patients with mirrored phenotype
Source: Front Neurol. 2023 Jan 4;13:1052341. doi: 10.3389/fneur.2022.1052341 (PMC9846158; doi:10.3389/fneur.2022.1052341)
Supplement: Supplementary file 1 [file Data_Sheet_1.docx]

**Supplementary Methods**

***Samples’ processing***

Genomic DNA was extracted by automated TECAN system (Freedom EVO) from venous peripheral blood using the NucleoSpin Blood method (Macherey-Nagel). Genomic DNA is isolated following lysis, which is achieved by incubating whole blood in a lysis solution. The appropriate conditions for DNA binding to the silica membrane of the corresponding NucleoSpin Blood columns are obtained by adding ethanol to the lysate. Wash steps effectively remove contamination, and pure genomic DNA is finally eluted in a slightly alkaline buffer.

***Genetic characterization tools***

This deletion was studied through ClinVar [1], a public genetic archive where relationships between genetic variations and human phenotypes can be assessed, with supporting evidence. Alleles described are mapped to reference sequences and reported according to the HGVS standard. ClinVar uses standard terms for clinical significance recommended by an authoritative source when available [1]. These standards include five terms for Mendelian diseases recommended by ACMG/AMP and terms for low-penetrance variants and risk alleles recommended by ClinGen [2].

***18F-FDG-PET***

*^18^*F-FDG-PET acquisition was performed according to published guidelines [3] and to the protocol described by Canosa et al [4].

***MRI***

MRI was performed by MRI HDXT Echo-Speed GE Signa 1.5 T.

***Diffusion tensor imaging (DTI)*** was acquired using a 2D single-shot spinecho planar imaging sequence and T1-weighted MRI using a standard 3D magnetization prepared rapid acquisition gradient-recalled echo (3D MPRAGE) pulse sequence at 1 mm isotropic spatial resolution

***DTI data pre-processing and analysis***.

The DTI datasets were corrected using FSL 5.0.8 (<http://www.fmrib.ox.ac.uk/fsl/>) and non-brain tissue was removed using the brain extraction tool [5]. We computed the apparent diffusion coefficients (ADC) from the diffusion-weighted and T2-weighted images and computation was conducted by using the 3dDWI2DT program from AFNI_17.0.18 (<https://afni.nimh.nih.gov)>.

**REFERENCES**

1. Clinvar: [https://www.ncbi.nlm.nih.gov/clinvar/variation/576569/?oq=SOD1[gene]+p.glu134del&m=NM_000454.5(SOD1):c.397GAA%5B1%5D%20(p.Glu134del)](https://www.ncbi.nlm.nih.gov/clinvar/variation/576569/?oq=SOD1%5bgene%5d+p.glu134del&m=NM_000454.5(SOD1):c.397GAA%5B1%5D%20(p.Glu134del))
2. Clingen: <https://clinicalgenome.org/>
3. Varrone A, Asenbaum S, Vander Borght T, Booij J, Nobili F, Någren K, et al. EANM procedure guidelines for PET brain imaging using [18F]FDG, version 2. Eur J Nucl Med Mol Imaging. 2009;36(12):2103–2110. doi: 10.1007/s00259-009-1264-0.
4. Canosa A, Calvo A, Moglia C, Vasta R, Palumbo F, Solero L, et al. Amyotrophic lateral sclerosis with SOD1 mutations shows distinct brain metabolic changes. Eur J Nucl Med Mol Imaging. 2022;
5. Smith SM: Fast robust automated brain extraction. Human brain mapping 2002, 17(3):143-155.
